# Voluntary upregulation of heart rate variability through biofeedback is improved by mental contemplative training

**DOI:** 10.1038/s41598-019-44201-7

**Published:** 2019-05-27

**Authors:** Boris Bornemann, Peter Kovacs, Tania Singer

**Affiliations:** 1 Department of Social Neuroscience, Max Planck Institute for Human Cognitive and Brain Sciences, Stephanstr. 1a, 04103 Leipzig, Germany; 2 Leipzig University Medical Center, IFB Adiposity Diseases, University of Leipzig, Liebigstr. 21, 04103 Leipzig, Germany; 3 Social Neuroscience Lab, Max Planck Society, Philippstr. 13, Campus Nord, Haus 5, 10099 Berlin, Germany

**Keywords:** Behavioural genetics, Autonomic nervous system, Human behaviour

## Abstract

Regulation of the parasympathetic nervous system, indexed through high frequency heart rate variability (HF-HRV), is indicative of physical and psychological health. However, little is known about the trainability of this capacity. We investigated the effects of a 9-month mental training program (the ReSource Project; n = 298) on voluntary HF-HRV upregulation, assessed with a novel biofeedback procedure. The program consisted of attentional, interoceptive, socio-affective and socio-cognitive training elements, all of which potentially influence parasympathetic regulation. Based on known links between oxytocin and parasympathetic activity, we also explored the relationship of HF-HRV upregulation to the oxytocin receptor system. We found that HF-HRV during the biofeedback session increased after 3 months of training, concomitant with prolonged respiration cycles. Breathing-controlled changes in HF-HRV upregulation, indicative of improved parasympathetic control, were significantly increased after 6 months of training. Homozygous risk allele carriers (AA) of the oxytocin receptor gene polymorphism rs53576 showed initially lower parasympathetic control, but fully compensated for their initial deficits through the training. No changes were found for HF-HRV at rest. Our data demonstrate that a mental training intervention extending over several months can increase the capacity for voluntary regulation of HF-HRV, with important implications for improving individual and societal health.

## Introduction

Many of the brain-body interactions underlying socio-affective processes implicate the body’s largest parasympathetic nerve: the vagus^1–5^. Activity of the vagus nerve can be measured non-invasively by quantifying the power of high frequency heart rate variability^6^ (HF-HRV). High frequency oscillations in heart rate are largely caused by the waxing and waning of the vagus’ slowing influence on the sinoatrial node. Specifically, the slowing influence of the vagus on the heart increases during exhalation and decreases during inhalation^7^. Thus, the distance of peaks and troughs in the oscillation of the heart rate indicates vagal activity.

Incidental upregulation of parasympathetic (vagal) activity, indexed by increases in HF-HRV, has been linked to the regulation of attention^8^ and emotion^9^, positive social engagement^10^, pro-social emotions, such as compassion^11–13^, and altruistic behaviour^14,15^. Recently, we have demonstrated that the ability to voluntarily increase HF-HRV predicts altruistic behaviour across a wide range of tasks^16^. This finding is in line with the role of the vagus in the mammalian care-system^17,18^, that is, the biological system underlying care-giving behaviour toward offspring and beyond. This system has been proposed as a biological basis of human altruism^19,20^. The neuropeptide oxytocin is central to this system. The vagus is densely populated with oxytocin receptors in rodents^19,21–26^ and intranasally administered and naturally secreted oxytocin increases HF-HRV in humans^27–30^. This further substantiates the role of the vagus in the care-system. Learning to voluntarily increase HF-HRV through biofeedback also has beneficial effects for diverse medical and psychopathological conditions such as asthma^31,32^, cardiovascular disease^33^, depression^34^, and PTSD^35^. To summarise, the increased parasympathetic tone associated with upregulated HF-HRV is marked by physiological and psychological quiescence which is conducive to regeneration of the organism and also facilitates positive social engagement and care-giving behaviours^20,36–42^.

Despite the importance of voluntary HF-HRV control for individual and societal health, little is known about its malleability. Biofeedback that explicitly targets voluntary HF-HRV control has been found to be effective^31–35^. It is, however, also conceivable that interventions using other self-regulatory methods to foster physiological and psychological well-being will also impact the ability to regulate HF-HRV. Secular meditation-based training programs (also called contemplative mental training) such as the 8-week MBSR^43^ (Mindfulness Based Stress Reduction) or MBCT^44^ (Mindfulness Based Cognitive Therapy) have gained attention recently because of their apparent health-benefits from reducing stress^45^ to increasing well-being^46^ and emotion regulation capacities^47^. As with physical exercises, however, there are many different types of mental training. While some programs focus on present-moment attention or mindfulness, others focus on emotional capacities, and yet others on meta-cognitive awareness of thoughts or the self^48–50^. To investigate (a) if contemplative mental training can boost our ability to regulate parasympathetic activity and (b) if yes, which type of practice is most efficient in doing so, we engaged 335 people in a 9-month training study, the ReSource project^49^. The intervention was comprised of secularised classical meditation exercises and “Contemplative Dyads”, which are 10-minute mental practices done with a partner^51^. The training was split into 3 modules of 3 months each (for details of the training, see Methods section). Each module had a different focus. In the first module, called the “Presence” module, the two core practices assigned for daily practice were a body scan and a breathing meditation. In the body scan participants feel their way through their body by successively focussing on the sensations of their feet, legs, pelvis, and so forth up to their head. In the breathing meditation, participants pay attention to the sensations of their breath, returning to these sensations whenever the mind has strayed. The two other 3-month modules of the ReSource training were called “Affect” and “Perspective” and also targeted the cultivation of intersubjective skills with classical meditation and a Contemplative Dyad as daily core practices. The Affect module focused on increasing socio-emotional capacities such as gratitude, compassion, loving-kindness, and acceptance of difficult emotions via “loving-kindness meditation” and an affective-based dyadic exercise. The Perspective module focused on socio-cognitive abilities such as meta-cognitive awareness, understanding the self, and taking the perspectives of other people with a thought observation meditation and a Contemplative Dyad (for details, see Methods section).

**Figure 1:**
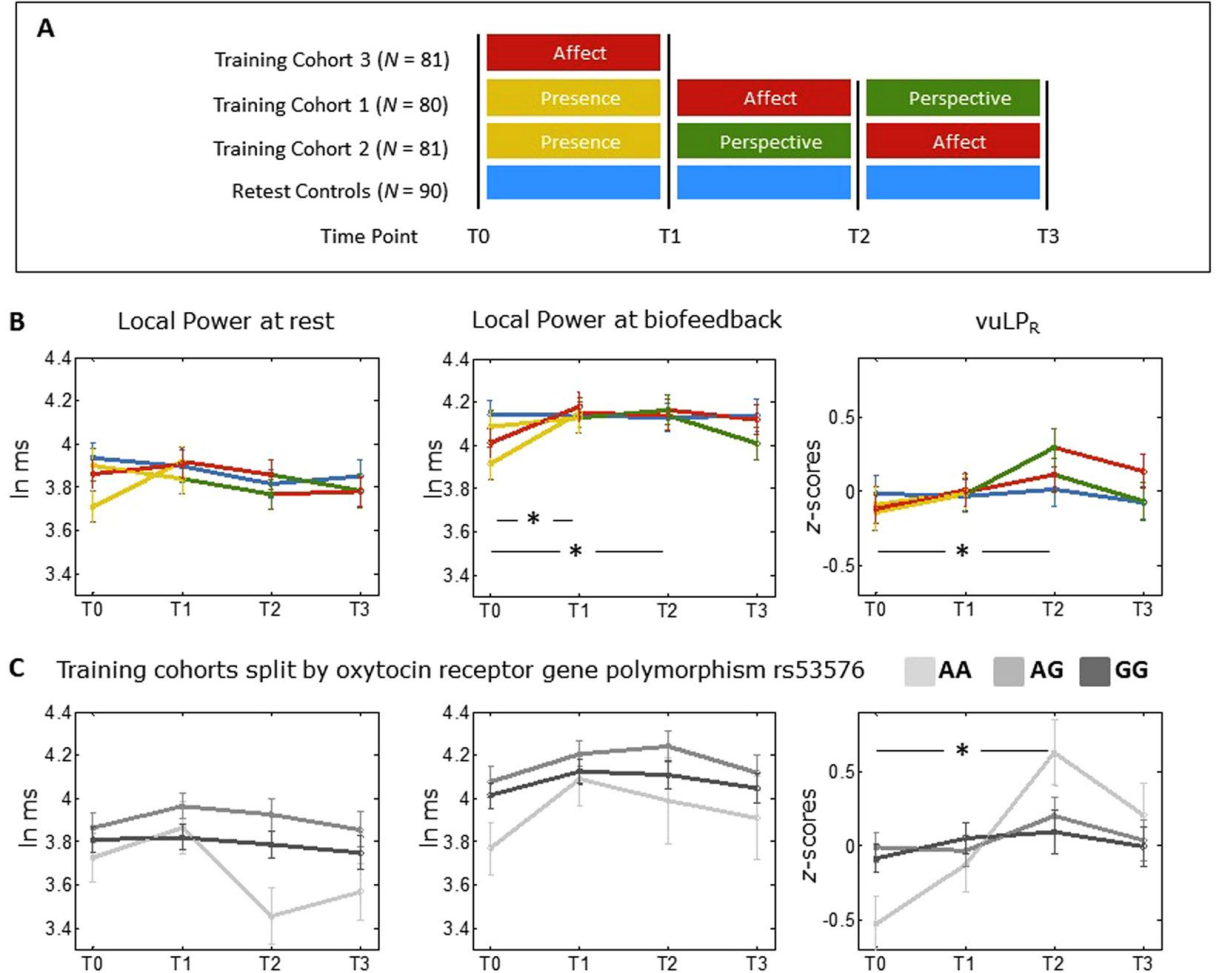
Changes in the three HRV parameters across the training. (**A**) Design of the ReSource project (reproduced with permission from^49^): Three training cohorts undergo different types of mental training. Measurements are taken at every time point (x-axis). Δt between the time points = 3 months, total training duration = 9 months. A retest control cohort completes all measurements but does not undergo any training. (**B**) Variation in the three HRV parameters across the training period. ln ms = logarithm of milliseconds. vuLP_R_ = voluntary upregulation of Local Power, controlling for changes in respiratory period. (**C**) Variation in the three HRV parameters in the trained cohorts across the training period, split by the oxytocin receptor gene rs53576 polymorphism. *p < 0.05, for the interaction between time and training from T0 to Tn, tested within a linear mixed model (see Methods). Error bars indicate standard error of the mean. Number of participants per timepoint are given in Table 1 (for Panel B) and Supplementary Table S2 (for Panel C). Note that the T0 data of panel B have been previously reported in^16^. Data of the graphs can also be found as tables in the Supplementary Material, Section F.

Two cohorts (n = 81 and n = 80) were initially trained over nine months (see Fig. 1, panel A). One cohort received the training in the order of Presence-Affect-Perspective, the other cohort in the order of Presence-Perspective-Affect. This counterbalanced design allows the use of the training groups as active control groups for each other, to detect effects which are specific to the module, rather than to mental training in general. A third cohort (n = 81) received only the Affect training (to control for the specific effect of the Presence module), and a retest control cohort (n = 90) did not receive any training but completed the same measurements at the same temporal distances. The intervention was delivered by a team of experienced meditation teachers and psychotherapists. Each module started with a 3-day residential retreat. While the participants were in silence, the teachers introduced them to the core practices and main topics of the respective modules. The retreat was followed by 13 weeks of module-specific training featuring weekly two hour sessions with teachers, and 30 minutes of daily home practice. Home practice was supported by a smartphone app and internet platform.

**Figure 2:**
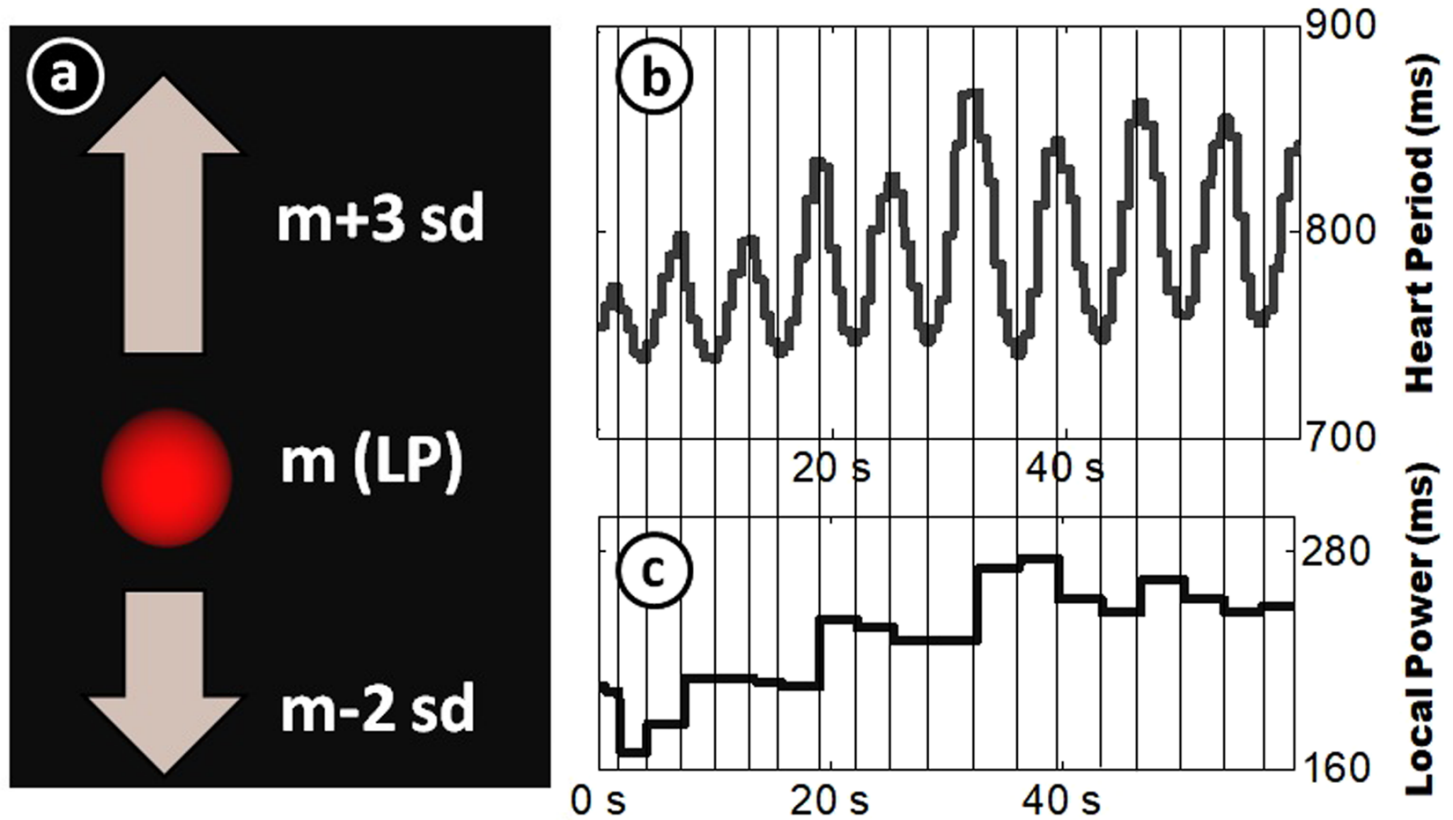
Biofeedback display and computation of Local Power. (Panel a) shows the display of the biofeedback task. A spinning ball is displayed on the screen. Its height is determined by Local Power (LP). (Panels b,c) illustrate the computation of LP: Shown is the heart period (HP) of a single participant over the course of 1 minute (Panel b). Every time the direction of the HP curve shifts (vertical lines), a new LP value (Panel c) is assigned, computed as the difference in HP to the previous shifting point. An average of the last two LP values is used for feedback. Figure reproduced with permission from^16^.

At four time points before and after each 3-month training module (i.e., at the beginning and after 3, 6, and 9 months), we investigated voluntary HF-HRV regulation using a newly developed biofeedback task^16^. In that task, participants were seated in front of a computer screen and asked to remain physically still. For five minutes we recorded HF-HRV at rest using an electrocardiogram. Subsequently, a spinning, three-dimensional ball was displayed on the screen (see Fig. 2). The altitude of this ball was determined by Local Power (LP), a measure of HF-HRV with high temporal resolution (for details, see^16^, and Methods section). Participants were asked to make the ball rise (which happened when Local Power increased), while remaining physically still. Participants were informed that the ball reflected some aspect of their “mental-bodily state”. However, no further information about this state was given, nor did we instruct any strategies to influence it (for the exact instructions see Supplementary Material, Section D).

We investigated three interrelated parameters of autonomic regulation: Local Power at rest (LP_Rest), Local Power at biofeedback (LP_BF), and voluntary upregulation of Local Power, controlled for breathing (vuLP_R_; for details, see Methods section). LP_Rest characterises the parasympathetic influence on the heart without any self-regulatory efforts. LP_BF characterises the parasympathetic influence on the heart during biofeedback. And vuLP_R_ characterises the ability to induce changes in parasympathetic activity, controlling for changes in the breathing pattern from rest to biofeedback, thus indexing the ability to regulate HF-HRV using other mechanisms than breathing control (such as upregulation of central vagal outflow).

Earlier studies have failed to find any effects of contemplative mental training on HF-HRV at rest^52–54^. Two studies, however, found that meditation (in the form of self-compassion training^52^ and a program focussing on emotion regulation^53^) leads to more situation-adequate adaptations of HF-HRV, that is, lower HF-HRV in stressful situations (vagal withdrawal) and higher HF-HRV during recovery from a stressor (vagal augmentation). Accordingly, we expected to find effects of the training not during LP_Rest, but during the two parameters indexing regulatory capacity (LP_BF and vuLP_R_). We assumed two principal mechanisms to facilitate these changes: Improvements in interoception (inner body sensing) and training of socio-emotional and socio-cognitive capacities. Interoceptive information partially travels from the body to the brain via the vagus nerve^3^. Improved interoception may thus affect vagal regulation. Additionally, continuous monitoring of bodily activity may make participants more aware of ways to use breathing to influence other physiological processes (e.g., change cardiac activity through breathing). The focus on interoception is particularly strong in the Presence module. However, the Affect and the Perspective modules also require participants to ground awareness in body sensations to focus on the present moment. Earlier studies have found that all three modules improve interoception^55^. Accordingly, we hypothesize that all modules will improve voluntary vagal regulation.

Previous research has established that vagal regulation underpins social engagement^2,20,56^, particularly, caregiving and altruistic behaviours^14–16^. As the Affect and the Perspective modules aimed at training these propensities, we hypothesized that they will also improve vagal regulation in the biofeedback task. As the biofeedback task itself does not involve social cues, though, this hypothesis rests on the assumption that the internal regulation schemes acquired during social training transfer to non-social situations. For instance, participants might be more benevolent with themselves during the task, promoting a more relaxed and thus parasympathetic state. Generally viable routes to vagal activation, such as voluntary relaxation^57^ and slower breathing^58^, might also be more effective in participants who have implicitly learned to activate the vagus through socio-affective training. This hypothesis would receive support from the data if the Affect and the Perspective module (which focus on social skills and incidentally also train interoception)^55^ prove to be more effective than the Presence module (which focuses primarily on interoception) in enhancing biofeedback performance. If they are less or only equally effective, we would not be able to disentangle the relative contributions of interoceptive and social training on changes in vagal regulation.

Based on the findings relating the oxytocin system to HF-HRV, we also explored how genetic differences in the oxytocin receptor system influence voluntary parasympathetic control. Specifically, we analysed the influence of the oxytocin receptor gene polymorphism rs53576. Homozygous carriers of the minor allele (A; hitherto referred to as ‘risk allele’) of rs53576 have been shown to exhibit lower interpersonal sensitivity^59–62^. For instance, risk allele carriers were shown to be less responsive to their toddler’s distress^59^ and had less pronounced cardiac reactions to infant’s crying sounds^60^. They show lower amygdala activation to social emotional stimuli, such as faces^61^, and show lower stress-buffering effects from the presence of a friend during a social stress-tests^63^. Assuming that this lowered interpersonal sensitivity is a function of diminished parasympathetic regulation, we explored whether AA-carriers perform worse in the biofeedback task. Because the training, especially the Affect and the Perspective module, is aimed at improving interpersonal skills, we furthermore assumed that it could diminish these initial differences between participants of different genotypes. Although motivated by previous theoretical reasoning and empirical data, these analyses had not been a primary goal of the ReSource project, as we did not know a priori which distribution of genotypes we would find in our sample and the ReSource project is lacking the sample size needed for rigorous genetic analyses. These additional analyses should thus be interpreted as purely explorative and in need of replication.

## Results

### Testing the specificity of the training modules

To determine whether the training modules had differential effects on HF-HRV and HF-HRV regulation we first compared two linear mixed models (for details see Methods). The first model included the four different conditions (Presence, Affect, Perspective, and no training) while a simpler nested model contained only training and no training (thus collapsing data across all training conditions). There were no differences in fit between these two models for any parameter (*p* > 0.30 for all). This indicates that the data were modelled equally well when only distinguishing between trained and untrained participants (regardless of the training type). We thus used this more parsimonious model for all subsequent analyses.

### Changes in HRV regulation

As mentioned in the introduction, we analysed two ECG-derived parameters to characterise regulation of heart rate variability. Raw Local Power during the biofeedback (LP_BF) quantifies the power of high frequency oscillations in heart rate. It is strongly related to other heart rate variability parameters (*r* = 0.85 to 0.98)^16^, such as spectral high frequency power^6^ and peak-to-trough respiratory sinus arrhythmia^64^. It is influenced both by breathing patterns and vagal activity^7,65^. In order to isolate vagal activity, we additionally analysed voluntary upregulation of Local Power, controlling for respiration (vuLP_R_), as done in previous research^16,52,66^. This parameter indexes regulation of cardiac vagal activity beyond breathing effects, suggesting regulation of central vagal outflow (for more information on these parameters, see Methods section).

After 3 months of training (from T0 to T1), LP_BF had increased in the Training Cohorts (TCs), but not in the retest control cohort (Fig. 1B), as indicated by a time * training interaction, *t*(738.5) = 2.48, *p* = 0.007, Cohen’s *d* = 0.240 (this and all subsequent *t*s refer to the interaction term within the linear mixed model). Trained participants had also increased in respiratory period (measured by a strain gauge), as indicated by a time * training interaction, *t*(736.1) = 2.34, *p* = 0.010, *d* = 0.295. Increases in respiratory period (i.e., breathing more slowly) correlated with concomitant increases in LP_BF, *r* = 0.296, *p* < 0.001. No statistically significant changes occurred in the parameter of voluntary parasympathetic control, vuLP_R_, *t*(767.0) = 0.89, *p* = 0.188. Together these results indicate that the first three months of the training increase the capacity to regulate heart rate variability and that this effect is largely achieved through slower breathing.

The increase in LP_BF in the training as compared to the control cohort remained significant after six months of training, *t*(746.3) = 2.29, *p* = 0.011, *d* = 0.254. Importantly, at this time point there was also a significant time * training interaction in respiration-controlled voluntary upregulation, vuLP_R_, *t*(780.8) = 1.77, *p* = 0.039, *d* = 0.288 (Fig. 1B). This indicates that participants achieved control of heart rate variability partially through non-respiratory mechanisms, such as upregulation of central vagal outflow. This increased parasympathetic control emerges after participants have completed the initial training phases as well as one of the interpersonal training modules.

There was a slight drop in biofeedback performance between month 6 and month 9 of the study, rendering the effects from T0 to T3 no longer significant for LP_BF, *t*(747.6) = 1.31, *p* = 0.09, or vuLP_R_, *t*(784.6) = 1.31, *p* = 0.13. We also investigated HF-HRV during the initial five-minute resting period. No changes over the training were found in Local Power or Local Power controlled for breathing, *p* > 0.10 for all tests. This indicates that the training specifically affects regulatory capacities, but not autonomous activity at rest.

Descriptively, the control cohort had higher initial values on LP_BF as compared to the training cohort. Although this difference was not statistically significant, *t*(297) = 1.66, *p* = 0.10, it still may have influenced the results. Ceiling effects may have impeded control cohort increases in LP_BF. Further, the changes in the training cohorts might be partially attributed to regression towards the mean^67^. To address this, we included into the model (a) the baseline (T0) value of LP_BF, (b) the interaction of time * baseline, and (c) the interaction of baseline * time * training. All these terms were significant, *p* < 0.001 for all tests, with a positive beta for the baseline value and negative betas for both interaction terms. This indicates that (a) participants with higher values at baseline also had higher values in later training phases, that (b) participants with higher baseline values showed less change, and that (c) baseline values affected the training-dependent changes over time. Within this model the time * training interaction for changes in LP_BF from T0 to T1 was still significant, *t*(721.6) = 2.362, *p* = 0.009. However, the interaction became marginal from T0 to T2, *t*(733.2) = 1.33, *p* = 0.092. This indicates that regression towards the mean and ceiling effects may have slightly inflated the observed training-dependent changes in LP_BF. When doing the same control analyses for LP_Rest and vuLP_R_ the pattern of significance remained unaffected.

### Modulation of HRV by oxytocin receptor gene polymorphism

Based on the links between the vagus nerve and the oxytocin system, we explored the potential influence of the oxytocin receptor gene polymorphism rs53576 on regulatory ability at baseline (T0). Genotype frequencies for rs53576 were N = 38 (13.24%) AA carriers, N = 120 (41.81%) AG carriers, and N = 129 (44.95%) GG carriers. This distribution is in Hardy-Weinberg equilibrium, *χ*^*2*^ = 0.494, *p* = 0.52. We found a significant relationship between rs53576 alleles and voluntary parasympathetic upregulation, vuLP_R_, *F*(2,284) = 3.198, *p* = 0.042 (see Supplementary Fig. S3). Post-hoc independent sample *t*-tests revealed that vuLP_R_ was significantly lower in homozygous carriers of the risk allele (AA) than in AG carriers, *p* = 0.027, *d* = 0.411, or GG carriers, *p* = 0.014, *d* = 0.454. The difference between AG and GG carriers was not significant, *t*(247) = 0.335, *p* = 0.738, *d* = 0.043. No relationship was found between genotype and Local Power at rest, or LP_BF. The genetic influence thus seems to be specific to vagal regulation, but not resting vagal activity.

### Modulation of training effects by oxytocin receptor gene polymorphism

We explored whether homozygous risk allele carriers would profit more strongly from the mental training, particularly the socio-affective and socio-cognitive training components, which might help them to overcome their initial deficits in interpersonal skills. Indeed, among participants who received the training, homozygous carriers of the risk allele (AA) of rs53576 showed significantly stronger vuLP_R_ training effects than G-carriers (AG/GG), as indicated by a significant time * genotype interaction, from T0 to T2, *t*(566.1) = −2.30, *p* = 0.022, see Fig. 1, Panel C. By the end of the intervention (at T3), the three genotypes no longer differed significantly in vuLP_R_, *F*(2,140) = 0.234, *p* = 0.792. This indicates that homozygous risk allele carriers profit most strongly from the training and fully compensate their initial deficits in vuLP_R_. No gene-dependent trajectories were found for LP_BF or Local Power during the resting baseline, *p* > 0.10 for all tests, again suggesting that the influence of the rs53576 polymorphism is specific to voluntary vagal control.

There appears to be a slight drop in LP_Rest from T0 to T2 in the group of AA carriers. This drop was not significant, neither when tested as a time * genotype interaction from T0 to T2, *t*(534.1) = 0.691, *p* = 0.49, nor when tested as the effect of time within the AA group alone, *t*(222.57) = 0.98, *p* = 0.33, both tests two-sided. It is however noteworthy that this drop will have increased the effect size of gene-dependent change in vuLP_R_ at T2 reported above, given the mathematical dependence of LP_Rest and vuLP_R_ (see Methods).

## Discussion

The goals of this study were to investigate whether voluntary upregulation of HF-HRV can be improved by contemplative mental training and if so, to determine which types of mental practices can bring about such increases in autonomic control. We used a novel biofeedback task to measure voluntary upregulation of HF-HRV, a cardiac marker of parasympathetic (vagal) control. We investigated this in the context of the ReSource project, a 9-month long mental training study consisting of three different 3-month training modules^49^. We showed that after 3 months of Presence training, a module focusing on present moment attention and interoception, participants indeed showed increased HF-HRV during the biofeedback task (measured by raw Local Power, a short-term measure of high frequency fluctuations in heart rate). No such changes were found for a retest control cohort. Increased regulation after these first 3 months of training, which included practices such as “breathing meditation” and “body scan”, was largely achieved by adopting slower breathing rates that are known to increase the influence of the vagus nerve on the heart^7,65,68^. We therefore suggest that the first months of training, which have a strong emphasis on body and breath observation, make participants more aware of ways to influence their bodily state through breath control. The ability to increase HF-HRV through slower breathing has been linked to symptom reduction in a variety of bodily and mental disorders, such as cardiovascular^33^ and pulmonary disease^31,32^, as well as depression^34^ and PTSD^35^, making these findings clinically relevant. It is noteworthy that participants in the control cohort showed descriptively slightly better biofeedback performance to begin with. These differences were, however, not significant and statistical control for baseline-dependency of change did not change the observed changes from T0 to T1, speaking for robust training-related increases. Still, future studies with less variance in the starting conditions may be helpful in replicating and validating our findings.

Interestingly, when using a breathing-controlled parameter of HF-HRV regulation (i.e., increases in Local Power from a resting baseline to the biofeedback, controlling for simultaneous increases in respiration cycle length), participants showed a significant increase after 6 months, that is, after completing either the Affect module or the Perspective module. These modules focused on socio-affective (compassion, loving-kindness) and socio-cognitive skills (perspective taking on self and others). In addition to classical meditation, these modules also included 10 minute contemplative dyadic exercises^51^. These findings suggest that the participants’ ability to increase the vagal influence of the heart has grown beyond breathing control. This could be attributed to the social nature of the training modules participants complete between month 3 and month 6. As the vagus is strongly implicated in social interaction^9,42,69^, intersubjective mental training may have had additional beneficial effects on its deliberate regulation. The vagus also plays an important role in the mammalian system underlying care-giving behaviour^18–20,70^. As the Affect module explicitly aims at fostering care-giving towards one-self (self-compassion)^71^ and others (pro-social behaviour), improvements in vagal upregulation might also be attributed to stronger activation of the care-system. Interestingly, however, our analyses revealed that there were no module-specific effects, but that it was rather training time which determined the observed increases on the two autonomic markers used here. Thus, the present data do not allow us to disentangle training-general from module-specific effects. We know from an earlier study^55^ that all of the investigated modules enhance the ability to feel the body (interoception, as measured by a heartbeat perception task). Thus, rather than the social nature of the Affect and Perspective training modules, it could also be an improved awareness of their own bodies which causes the observed enhancement in parasympathetic control. Future studies are warranted to disentangle the contributions of social and interoceptive training components on parasympathetic control. They could also strive to disentangle the effects of different training components such as silent retreats and weekly group sessions, as well as single meditation and dyadic practices on HF-HRV regulation.

All training effects were in a range of Cohen’s *d* = 0.240 and *d* = 0.295, which are conventionally labelled small effects^72^. However, this effect may play out in a lot of different physiological and psychological domains, given that HRV has been implicated in a wide range of outcomes from cardiac health, and emotion regulation to altruistic behaviour^9,10,73^.

The increases in HF-HRV control demonstrated after 6 months were no longer significant when tested 3 months later (at the end of the study). This unexpected finding may be due to the nature of the employed task. Whereas participants generally reacted with great interest to the biofeedback task in the initial measurement sessions, this interest may have declined through repetition, yielding changes in strategies or a lower involvement in the task. This may explain the slight drop of performance observable in the last measurement period. These fluctuations in interest in the task may be a limitation of the biofeedback task in repeated-measure designs. Apart from that, the data suggest that the task is sensitive to training-induced changes, rendering the novel biofeedback task a feasible tool for future research on voluntary autonomic regulation. An alternative explanation for the unexpected drop in HF-HRV control at T3 is that the training effects are volatile or that they characterize only a transient improvement. Investigation of participants’ performance at later time points (several months after the end of the training) may clarify this issue.

Finally, after having established that mental training can indeed have beneficial influences on voluntary parasympathetic regulation, we explored how this ability is modulated by the oxytocin system. The vagus nerve is densely populated by oxytocin receptors^19,21–26^ and intranasal administration of oxytocin has been found to increase HF-HRV in humans^27–30^. We thus explored whether individual differences on the oxytocin receptor gene polymorphism rs53576 modulated respiration-controlled HF-HRV regulation. We found that homozygous carriers of the risk allele (AA) of rs53576 showed a lower capacity for voluntary parasympathetic control at baseline. Previous studies have shown that AA-carriers exhibit lower interpersonal sensitivity^59–62^. Because relating to others is partially based on vagal regulation^2,9,15,69,74,75^ these two findings may be linked: The reduced social sensitivity of AA-carriers may also be partially rooted in a relative inability to regulate vagal activity. Furthermore, we found that the trajectories of training-related increases in autonomic control differ by genotype, with AA-carriers profiting most from the training. In fact, the increase in AA-carriers seems to drive the overall increases in the training cohorts. Possibly, their initial deficits may make the social exercises most fruitful for them, leading to stronger improvements than in G-allele carriers. Conversely, a ceiling effect may prevent G-carriers from increasing as much as homozygous A-carriers. Together, our explorative analyses and preliminary findings suggest a genetic basis for individual differences in parasympathetic control as well as its malleability through training. However, due to the relatively small sample sizes of the groups with an N of only 38 for AA carriers (and Ns of 120 and 129 for AG and GG carriers, respectively) these genetic analyses should be treated with great caution and future replication studies in bigger samples are clearly needed. It should also be noted that this genetic effect would not survive correction for multiple comparisons.

No effects of the training were found for Local Power at rest. This is in line with earlier studies investigating the effects of contemplative mental training on HF-HRV^52–54^. The only exception is a study by Tang *et al*.^76^. Notice, though, that this is the only study using normalised HF units instead of raw HF power, and that it uses a non-standard frequency band, 0.16–0.45, to determine the HF range. It thus seems that the training does not affect autonomic balance at rest, but rather the ability to induce changes therein. Both heart and breathing rhythm as well as the vagus nerve are under the control of evolutionarily old brainstem structures such as the pons and medulla oblongata^77,78^. Due to their vital role in preserving basic functions of the organism and their involvement in a large host of brain processes^79^, their basic activity may be slow to change. This may explain why it is hard to alter resting HF-HRV. The activity of those areas is, however, modulated by cortical activity, particularly by the medial prefrontal cortex^80^ (mPFC). This is probably how HF-HRV is affected through conscious effort^81^, such as the regulatory activity during biofeedback. We speculate that participants in our study have improved in the ability to exert a phasic influence of the mPFC on the brainstem, thus affecting HF-HRV. Such modulation is not tonic however, leaving resting HF-HRV unaltered. Future research with long-term practitioners of contemplative mental training (e.g., 15–40 years^82^) could clarify whether the modulatory effects become tonic, leading to permanently elevated levels of HF-HRV.

To summarise, we have shown that a mental training intervention increases the ability to voluntarily increase HF-HRV. This has implications for the use of such interventions in healthy and impaired populations. Mental training may prove particularly fruitful for people who exhibit altered or reduced vagal flexibility, as found in children at risk of psychopathology^83^ and people suffering from depression^84^ or social phobia^85^. Such programs may also be helpful for people suffering from psychotic symptoms, who have been found to benefit from increased HF-HRV control in a biofeedback study^86^. In addition, increased vagal control may have benefits for healthy individuals, as increases in vagal activity promote relaxation and restoration of the organism, thus increasing general well-being and resilience^87–90^. Furthermore, the ability to self-induce states of higher vagal activity may serve as a resource that allows individuals to more successfully reach out to other people in need, as suggested by previous studies^14–16^. Our results thus indicate that the investigated types of contemplative mental training may help build a healthier and more caring society.

## Methods

### Participants

Participants of the study were part of the ReSource Project, a large scale study on contemplative mental practice. Details about the recruitment procedure and randomised cohort assignment can be found in^49^, chapter 7. Briefly, all participants were mentally and physically healthy, had no regular meditation practice, and had not participated in a meditation retreat in the last 2 years. For the present study, physiological data of *N* = 298 participants (*n* = 251 in the training cohorts and n = 81 in a retest control cohort; 173 female, *M* age: 40.4; *n* = 287 with complete gene data) were available. Reasons for missing data are listed in the Supplementary Material, Section B. Participant characteristics for all study cohorts are listed in Table 1. We used data of all participants who had at least one time point in the study, as common for linear mixed models^91^. Note that the T0 data of this sample are identical to those used in Bornemann, *et al*.^16^. The present study also includes data from three subsequent time points (T1, T2, and T3) allowing the assessment of heart rate variability regulation through contemplative mental training over the course of the 9-month ReSource training. In addition, we explore how the oxytocin receptor gene polymorphism rs53576 affects parasympathetic regulation at baseline and as a result of training.

**Table 1:** Participant characteristics across the four time points.

	T0	T1	T2	T3
**Cohort 1** Presence - Affect - Perspective	n = 64 (33f), M age: 40.6 (9.0)	n = 76 (44f), M age: 41.5 (9.0)	n = 76 (42f), M age: 41.6 (8.6)	N = 73 (44f), M age: 41.4 (9.0)
**Cohort 2** Presence - Perspective - Affect	n = 76 (45f) M age: 41.2 (9.8)	n = 70 (43f), M age: 41.1 (9.7)	n = 73 (46f), M age: 41.3 (9.7)	n = 74 (44f), M age: 40.2 (9.7)
**Cohort 3** Affect	n = 77 (47f) M age: 40.3 (8.8)	n = 70 (41f), M age: 40.7 (9.0)		
**Retest Control Group** No Training	n = 81 (48f), M age: 39.6 (9.3)	n = 76 (47f), M age: 40.7 (9.0)	n = 82 (48f), M age: 39.7 (9.4)	n = 74 (47f), M age: 39.9 (9.5)
**All samples combined**	n = 298 (173f), M age: 40.4 (9.2)	n = 292 (175f), M age: 40.7 (9.2)	n = 231 (137f), M age: 40.8 (9.2)	n = 221 (131f), M age: 40.8 (9.4)

f = females; m age = mean age (standard deviation in parenthesis). The T0 data have been reported on in^16^.

### Ethics

The study was approved by the Research Ethics Committee of the University of Leipzig, number 376/12-ff and the Research Ethics Committee of the Humboldt University in Berlin, numbers 2013-02, 2013-29, and 2014-10. The study was registered with the Protocol Registration System of ClinicalTrials.gov under the title “Plasticity of the Compassionate Brain” with the ClinicalTrials.gov Identifier: NCT01833104. All methods were carried out following the relevant guidelines and regulations. All participants gave informed consent prior to the study.

### Gene assessment

Genomic DNA was extracted from blood, drawn after the T0 measurement, using the Quick Gene DNA whole blood Kit (Kurabo, Japan). Genotyping of the rs53576 was performed using the TaqMan SNP Genotyping assay (Applied Biosystems; C___3290335_10). The TaqMan genotyping reaction was amplified on an Applied Biosystem 2720 Thermal Cycler 95 °C for 15 min, and 95 °C for 15 sec, and 60 °C for 1 min, for 40 cycles) and fluorescence was detected by using the Applied Biosystem 7500 Real-Time PCR systemon (Thermo Fisher Scientific Inc.). To assess genotyping reproducibility, a random ~5% selection of the sample were re-genotyped for all SNPs; all genotypes matched initially designated genotypes.

### ReSource training

The overarching goal of the ReSource Project is to improve subjective well-being, mental and physical health, as well as compassion and intersubjective skills. To achieve these goals, the ReSource training aims to cultivate several attentional, socio-cognitive, and socio-affective processes taught in three separate modules called Presence, Affect, and Perspective. Each module was delivered over a period of three months, beginning with a 3-day silent retreat (silent except for the teacher instructions and explanations) and including 13 weekly group sessions of 2 hours and 30 minutes of daily practice. The training was facilitated by a team of experienced meditation teachers and psychotherapists and supported by an online-platform and a smartphone app. The three modules are described in detail in^49^, chapters 2 and 3.

Each module of the program has two core exercises, which participants were asked to practice 5 times a week, in addition to the weekly group sessions. Briefly, in the Presence module, participants learned to direct their attention to their present moment experience. The two core practices were Breathing Meditation and Body Scan. In the Breathing Meditation^92^, participants focused their attention on the sensations of their breathing, returning to these sensations whenever the mind had strayed. In the Body Scan^43,93^, participants attuned to bodily sensations, systematically and slowly moving the focus of attention from feet to head or head to feet, allowing sensations from all regions of the body to arise in the mind. In the Affect Module, participants practiced generating an attitude of kindness, care, and compassion for themselves and others. They learned how to approach difficult emotions with acceptance and to nurture prosocial motivations. The core practices in this module were a Loving-Kindness Meditation and a contemplative dyadic exercise referred to as the Affect Dyad. The Loving-Kindness Meditation^94^ involved connecting to an attitude of benevolence and care towards one self and others by using internally repeated phrases expressing these intentions and related imagery. The dyadic exercise can be conceptualized as a verbalized meditation. Participants sit facing each other. One of the participants shares a recent situation that evoked difficult emotions^95–97^ as well as a situation that evoked gratitude in them^98–100^. Focus is paid to how these situations affected their body. The other listens mindfully before they switch roles. In the Perspective Module, participants engaged in meta-cognition and in cognitive perspective-taking on one’s self and others. The core exercises in this module were an Observing-Thoughts Meditation and a dyadic exercise called the Perspective Dyad. In the Observing-Thoughts meditation^43,101,102^, participants practiced the observation of thoughts as mental events. They were taught to observe thoughts as natural events, comparable to sounds or bodily sensations. In the Perspective dyad, one partner chooses a recent situation and then randomly draws a previously identified personal “inner part” (a personality aspect in the framework of the Internal Family System)^103,104^. The participant then describes the chosen situation from the perspective of that inner part, thereby practicing perspective taking on one’s self. The partner listens mindfully and tries to identify the inner part whose perspective the other is speaking from, thereby training perspective-taking on others or Theory of Mind^105^.

Participants were required to log in on the online platform via computer or smartphone for each practice, allowing us to assess practice frequency and duration.

### Study design

The ReSource study was designed to test effects of different types of mental training on a large array of behavioural, subjective, physiological and brain measures (see^49^, chapter 8). An overview of the design is given in Fig. 1A. Two cohorts underwent all three training modules in counterbalanced orders, that is, Presence-Affect-Perspective and Presence-Perspective-Affect. These serve as active control cohorts to each other, allowing us to compare the effects of the Affect module to those of the Perspective module (by comparing test scores at T2). It also makes it possible to compare the efficacy of different training orders (when comparing test scores after the entire training). An additional cohort underwent only the Affect training, allowing us to ascertain specific effects of a module with strong interoceptive focus (Presence) in comparison to a socio-affective training module (Affect). A retest control cohort underwent no training but completed the same measurements as the training cohorts. This allowed us to test for training general effects, not differentiating for training type. In the particular study reported here, we did not have any specific hypotheses about order effects, nor about differential effects of Perspective and Affect training. Rather, we were interested in whether the training has effects at all, and if so, if any module is more effective than the other. We thus adopted the modelling approach described below (see Model selection) that first tests for module specificity and then collapses all modules in case no specificity is revealed.

### Physiological recordings

Heart rate and respiratory period were assessed throughout the resting measurement and the biofeedback task. For heart rate measurement, a standard Lead-II electrocardiogram was acquired by placing disposable Ag/AgCl Electrodes on the right mid-clavicle, left ribcage, and left outer clavicle. To measure respiration, a strain gauge was attached around the torso of participants at the level of the lower ribs, capturing both chest and abdominal breathing. Signals were recorded at 1000 Hz using a Biopac® (BIOPAC Systems Inc., Santa Barbara, CA) MP150 data acquisition system and the software AcqKnowledge 4.3. Data were processed and cleaned for artefacts (see Supplementary Material, Section C) with ARTiiFACT^106^ and custom MATLAB scripts (available on request).

### Resting measurement

For assessment of resting HF-HRV, participants were asked to just sit still and “not think of anything in particular” for 5 minutes, as is common for resting HF-HRV assessment^6^. The resting measurement was always done before the biofeedback task.

### Biofeedback task

The biofeedback task used to derive a measure of voluntary HF-HRV upregulation is described in more detail in^16^. Briefly, a red, spinning, three-dimensional ball was presented on the screen (see Fig. 2). Its vertical position was determined by a short-term estimate of HF-HRV (Local Power, see below). Participants were asked to make the ball go up by bringing themselves into a mental-bodily state that would be mirrored in the altitude of the ball. They were not told how to achieve that state but to experiment for themselves to find a state that is conducive to making the ball go up. Exact instructions can be found in Supplementary Material, Section D.

### HRV Parameters

In the last decades, several methods have been developed to estimate vagal activity from an electrocardiogram^6^. All of them aim to quantify the variability of the heart period (the time from one heart beat to another), which is caused by the activity of the vagus nerve. The vagus has a tonic slowing influence on the heart. This influence increases during exhalation leading to longer heart periods, whereas it decreases during inhalation, leading to shorter heart periods^7^. These increments and decrements in heart period, which occur along with respiration, are the main source of variability in heart period (or heart rate, which is the inverse of heart period). The difference between heart period during exhalation and inhalation is indicative of vagal activity^6^. A widely used measure to quantify this is spectral high-frequency HRV. To obtain this measure, a series of heart periods (typically from recordings of at least 1 min in length) is subjected to a Fourier transformation to derive oscillations in different frequency bands. The frequency band which corresponds to adult human respiration (0.15–40 Hz) is dubbed ‘high frequency heart rate variability’, because it contains higher frequencies than those typically caused by other (e.g., sympathetic) influences on the heart. The power of this frequency band (its amplitude) is taken to indicate vagal activity. Another common measure is peak-to-trough Respiratory Sinus Arrhythmia (pt-RSA)^64^. Respiratory Sinus Arrhythmia generally denotes the phenomenon described above, that is, the close coupling of fluctuations in heart period with the respiration cycle. In pt-RSA a respiratory recording is used along with the heart period data. Pt-RSA is computed as the maximum heart period during exhalation minus the minimum heart period during inhalation. As the distribution of HRV measures is highly skewed, they are typically transformed by using their (natural) logarithm.

In this study, we used an HRV measure called Local Power (explained in Fig. 2; and also see^16^). Local Power (LP) was specifically developed for biofeedback purposes, as it can be computed online with high temporal resolution. It quantifies oscillations in heart period data by computing the difference of a trough to a successive peak in the heart period series or vice versa. Heart period data are continuously fed to the biofeedback program. Whenever there is a turning point in the data (a change from acceleration in heart period to deceleration or vice versa) the heart period at this point is saved. When the next turning point appears, the difference to the previous turning point is computed and fed back as the current estimate of vagal influence on the heart. Depending on participant characteristics (particularly breathing rate), LP can be fed back to participants with an average temporal resolution of 2.46 s (SD = 0.64 s). A previous study^16^ has shown that LP is highly correlated to both pt-RSA (*r* = 0.98) and spectral HF-HRV (*r* = 0.85). These measures would have had several disadvantages compared to LP when used for biofeedback. Pt-RSA has only about half the temporal resolution, meaning feedback would have been slower. Furthermore, it relies on respiratory data, the online assessment of which had proven to be error prone in pilot experiments. Spectral HF-HRV requires about 1 min of heart period data to be accurate^6^. Furthermore, it makes assumptions about the breathing frequency to be between 0.15 and 0.40. This range may be easily violated if participants adopt particularly low or fast breathing rhythms to influence their physiological state during biofeedback. We used LP in both the resting and the biofeedback condition to assure comparability.

It has been found that slower breathing enhances the vagal influence on the heart, as the effect of respiratory gating on the sinus node is more pronounced during slower breathing^7,107,108^. Thus, HRV is increased by slower breathing. Although slower breathing may eventually enhance parasympathetic activity^109^, initial changes in HRV are often only due to the gating effect of respiration on vagal transmission to the heart, and not to changes in central vagal outflow^65^. Thus, it has been recommended to control for respiratory effects when making inferences on changes in vagal activity from one condition (e.g., rest) to another (e.g., biofeedback)^65^.

Three HRV parameters are analysed in the current manuscript:

1. Local Power at Rest (LP_Rest). This parameter indexes short term fluctuations in heart rate, which are mostly due to central vagal outflow as well as the breathing pattern.
2. Local Power at biofeedback (LP_BF). This is the same parameter as (1), but during the biofeedback task. It thus indexes participants’ ability to induce oscillations in heart rate by both changes in central vagal outflow and breathing pattern.
3. Voluntary upregulation of Local Power, controlled for respiration (vuLP_R_), was computed (as in^16^) as the standardised residual of the linear regression of [RSP_BF–RSP_BF] on [LP_BF–LP_Rest], where RSP denotes mean respiratory period. This parameter indexes changes in heart rate oscillations from rest to biofeedback, which cannot be attributed to changes in respiration cycle length. It is thus a purer measure of the ability to increase central vagal outflow (voluntary parasympathetic control).

### Model selection

To investigate changes in HF-HRV and HF-HRV regulation over time, we used linear mixed models, fit using *R*, version 3.1.2, and the modelling package *lme4*^110^. The model that was used to investigate differential change between the training cohorts and the retest control cohort included time, training, and their interaction as fixed effects and subject-specific intercepts as a random effect. Models including random slopes for training effects in addition to random intercepts were unidentifiable due to large eigenvalue ratios or did not converge due to non-positive Hessian matrices. Training type was first included as a variable with four categories coding for the four different conditions (Presence, Affect, Perspective, and no training). This model was tested against the simpler nested model that only tests for overall training effects (no training vs. training).

There were no significant differences in model fit between the two models for any of the HRV parameters as revealed by log likelihood chi-square tests, *p* > 0.30 for all tests. This indicates that the training types did not differ in their effect on resting or regulated HRV and that the data are modelled equally well by a simpler model that only distinguishes between trained and untrained participants. We thus used this more parsimonious model for all analyses. The main test of differential changes between the TCs and the RCC was the time*training interaction. We report *contrasts*, which probe the significance of changes in the DV from the initial value in the DV (at T0) to each of the three time points during the training (T1, T2, T3). This allowed us to test for changes in the DV that are not uniform over the entire training period (e.g., a DV that peaks at one time point and then stagnates or deteriorates). Within each model, contrasts were not corrected for multiple comparisons, which is justified because in the multilevel framework used here, estimates are “shrunk” toward a common mean using partial pooling, thus correcting for the increased risk of false positives typically incurred by multiple comparisons without compromising power^111^. The test statistic of the contrasts follows a Student’s *t*-distribution. One-tailed *t*-tests were used for contrasts for LP_BF and vuLP_R_ because we specifically hypothesised *increases* in these parameters through the training rather than *decreases*.

### Control variables

We repeated all analyses using age, sex, and BMI as control variables, because they potentially influence HF-HRV^112,113^. The pattern of significance remained the same in all analyses except for the effect of the rs53576 polymorphism on parasympathetic regulation at baseline (T0). The *F*-Test became marginal, *F*(2,280) = 0.08. The individual contrasts between the AA-carriers and the AG- and GG- carriers, however, remained significant, *p* = 0.049 and *p* = 0.027, respectively.

It has previously been reported that nicotine consumption reduces parasympathetic activity. We thus investigated the influence of smoking on the HRV parameters. There was a fairly low amount of smokers in our sample (11.3%) with an average cigarette consumption of 15.1 cigarettes/week (among the smokers only; *SD* = 13.18). We detected no influence of smoking on any of the HRV parameters, *p* > 0.4 for all tests, nor did the amount of cigarettes per week correlate to any of the parameters, *p* > 0.05 for all tests. There were also no interaction effects of smoking with the training effects, *p* > 0.15 for all tests.

## Supplementary information

Supplementary Material

## Data Availability

All data are available upon reasonable request from the Department of Social Neuroscience of the Max Planck Institute for Human Cognitive and Brain Sciences.
