# Supplementary material for: Voluntary upregulation of heart rate variability through biofeedback is improved by mental contemplative training
Source: Sci Rep. 2019 May 27;9:7860. doi: 10.1038/s41598-019-44201-7 (PMC6536553; doi:10.1038/s41598-019-44201-7)
Supplement: Supplementary file 1 — Supplementary Material [file 41598_2019_44201_MOESM1_ESM.docx]

Supplementary Material to

**Voluntary upregulation of heart rate variability**

**through biofeedback is improved by mental contemplative training**

*Boris Bornemann, Peter Kovacs, & Tania Singer*

A – Overview of the ReSource training protocol
B – Reasons for missing data per cohort and time point
C – Artifact treatment
D – Instructions in the biofeedback task
E – Respiration during rest and biofeedback
F – Modulation of LP_Base, LP_BF, and vuLP_R_ by oxytocin receptor gene polymorphism rs53576 at the beginning of the study (T0)
G – Sample size and genotype frequency in the training groups by time point (supplement to Figure 1 of the manuscript)
F – Data of Figure 1 as table

**A – Overview of the ReSource training protocol**


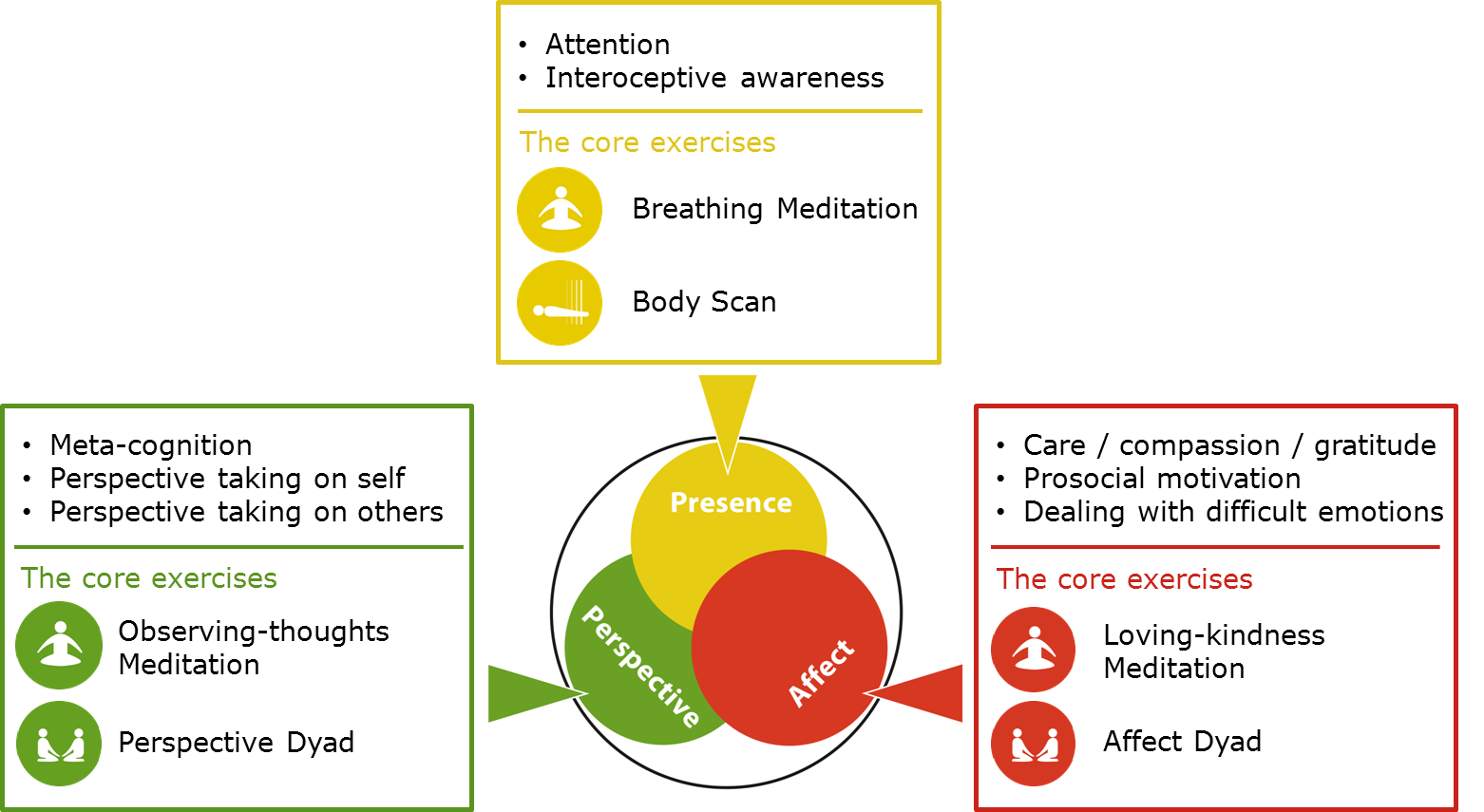


**Figure S1. The ReSource model.** The three modules of the ReSource training: Presence, Affect, Perspective. Bullet points name the core processes targeted in the respective training module. The two core exercises, named below, are practiced for a total of 30 minutes a day by participants of the training. Details about the training are found in ^1^, chapters 2 and 3. Figure reproduced with permission from ^1^.

**B - Reasons for missing data per cohort and time point**

Participants of the ReSource Project were recruited in a multi-step procedure described in detail in Singer, et al. ^1^, chapter 7. Briefly, a total of 4739 people applied for participation. Out of these, 332 were selected based on several inclusion criteria such as good mental and physical health, no intensive experience with meditation, and ages between 20 and 55. They were allotted to four different cohorts that were matched with regard to age, sex, and various demographic and psychological variables (see Singer, et al. ^1^, chapter 7). In the training cohorts (TCs) there were n = 80 (TC1), n = 81 (TC2), and n = 81 (TC3) participants at the beginning of the study. N = 90 participants were in the retest control cohort (RCC). Before each measurement time point, some participants dropped out for various reasons that are listed in ^1^, chapter 7.2. Reasons included time constraints, medical reasons, or feelings of discomfort. In the following table (Table S1), these participants are listed in the row “dropout”. Note that the time point of dropout indicated in the book ^1^ may differ from the time point indicated here. This is because the numbers in the book indicate a participant as dropped out at the time point where no data at all were present for the participant, whereas the table here marks a participant as dropped out if he or she left the study before HRV measurements. The remaining participants either could not be scheduled for a measurement session (“scheduling problems”) or technical problems such as excessive amounts of artifacts (see section B) or recording failures made their data unusable (“technical reasons”).

|  | dropout | | | | scheduling problems | | | | technical reasons | | | | tested n | | | | |
| --- | --- | --- | --- | --- | --- | --- | --- | --- | --- | --- | --- | --- | --- | --- | --- | --- | --- |
|  | TC1 | TC2 | TC3 | RCC | TC1 | TC2 | TC3 | RCC | TC1 | TC2 | TC3 | RCC | TC1 | TC2 | TC3 | RCC | Σ |
| T0 | 1 |  |  | 4 | 2 | 2 |  | 1 | 13 | 3 | 4 | 4 | 64 | 76 | 77 | 81 | 298 |
| T1 | 2 | 4 | 3 |  |  | 1 | 4 | 3 | 1 | 6 | 4 | 7 | 76 | 70 | 70 | 76 | 292 |
| T2 | 1 | 1 |  | 2 |  |  |  | 2 |  | 3 |  |  | 76 | 73 |  | 82 | 231 |
| T3 | 4 | 1 |  | 3 |  |  |  |  |  | 1 |  | 7 | 73 | 74 |  | 74 | 221 |

**Table S1***.* Overview of dropout and dropout reasons. TC1 = Training Cohort 1, TC2 = Training Cohort 2, TC3 = Training Cohort 3, RCC = Retest Control Cohort.

**C – Artifact treatment**

Interbeat interval (IBI) data were extracted from raw ECG using BIOPAC Acqknowledge’s (BIOPAC Systems Inc., Santa Barbara, CA) *Find Rate* function. Interbeat interval (IBI) data were then analyzed using Artiifact^2^. In an initial step, all data were subdued to automatic correction (batch processing) to detect artifacts. Cases for which at least one artifact was detected were checked manually by research assistants who were blind to the hypotheses. Research assistants went back to the original ECG data using BIOPAC Acqknowlegde when in doubt about the correct labeling of a specific IBI as an artifact. Artifacts were then corrected by using cubic spline interpolation. Cases for which 6 or more successive IBIs were marked as an artifact were excluded from the analysis for that time point, because cubic spline interpolation then resulted in flat lines (stationary IBIs) which would markedly influence HF-HRV quantification^3^. Corrected IBI files were overlain with the original IBI data using a custom made MATLAB script and screened again by the first author of the study to assure that all artifacts had been spotted and properly corrected.

**D - Instructions in the biofeedback task**

[Section reproduced from Bornemann, et al. ^4^ .]

The following instructions were read out by the experimenter prior to the biofeedback task:

*In the following task, we ask you to put yourself into a certain mental-bodily state. Your state is mirrored in the height of a ball on the screen. The more you are in that state, the higher the ball will rise.*

*Try to find out how you can induce the state in yourself. Please remain physically still, with your feet on the ground and hands in your lap.*

*Your task is to make the ball rise and keep it up as long as possible. Try to find out which mental-bodily state is most suitable for you to accomplish that. The task lasts for 5 minutes. Please stay with the task for the entire time of the experiment and try not to be frustrated if you have difficulties influencing the height of the ball.*

Original German Version:

*In der folgenden Aufgabe sollen Sie sich selbst in einen bestimmten körperlich-geistigen Zustand versetzen. Ihr Zustand wird in der Höhe eines Balls auf dem Bildschirm widergespiegelt. Je mehr Sie sich in dem Zustand befinden, umso höher wird der Ball steigen.*

*Versuchen Sie selber herauszufinden, wie Sie den Zustand in sich herbeiführen können. Bleiben Sie dabei körperlich bitte völlig ruhig, mit den Füßen auf dem Boden und den Händen im Schoß.*

*Ihre Aufgabe ist es also, den Ball hochsteigen zu lassen und so lange wie möglich dort zu halten. Finden Sie heraus welcher körperlich-geistige Zustand dafür für Sie am besten geeignet ist. Die Aufgabe geht insgesamt 5 Minuten. Bleiben Sie bitte während der gesamten Zeit des Experiments bei der Aufgabe und lassen Sie sich möglichst nicht davon frustrieren, wenn es Ihnen schwer fällt, die Flughöhe des Balls zu beeinflussen.*

**E – Depictions of respiration period during rest and biofeedback across all time points**


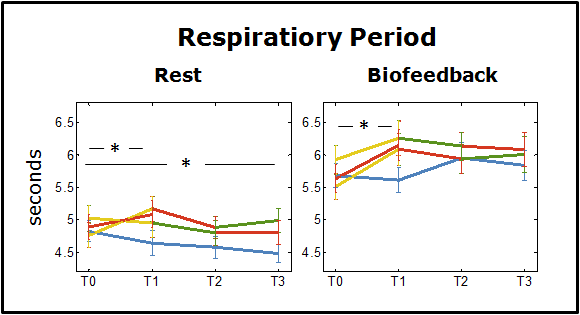


**Figure S2. Changes in respiratory period across the training during rest and biofeedback.** X-Axis displays the 4 time points of the study. Δt between the time points = 3 months, total training duration = 9 months. Yellow = Presence training, green = Perspective training, red = Affect training, blue = no training. See Fig.2, panel A, for an overview of the study design. * p < .05, for the interaction between time and training. Error bars indicate standard error of the mean.

**F – Modulation of LP_Base, LP_BF, and vuLP_R_ by oxytocin receptor gene polymorphism rs53576 at the beginning of the study (T0)**


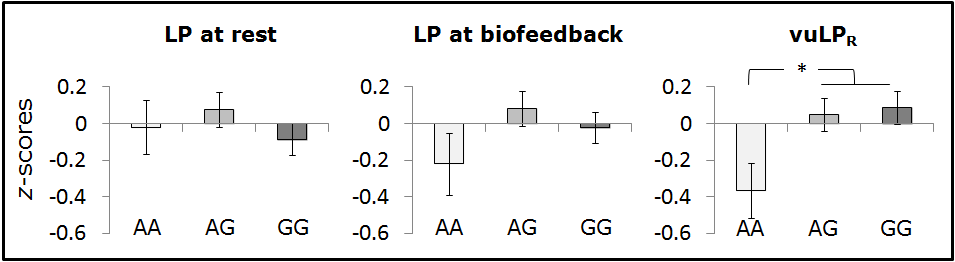


**Figure S3. Relationship of the oxytocin receptor gene rs53576 polymorphism to the three HRV parameters.** LP = Local Power; vuLP_R_ = voluntary upregulation of Local Power, controlling for changes in respiratory period. * p < .05. Error bars indicate standard error of the mean.

**G – Sample size and genotype frequency in the training groups by time point (supplement to Figure 1 of the manuscript)**

|  | **T0** | **T1** | **T2** | **T3** |
| --- | --- | --- | --- | --- |
| **AA** | 24 | 23 | 16 | 16 |
| **AG** | 87 | 91 | 64 | 62 |
| **GG** | 98 | 97 | 65 | 65 |

**Table S2.** Shown are the numbers of participants in the trained cohorts by genotype (AA/AG/GG) of the oxytocin receptor polymorphism rs53576 by time point (T0 = baseline, T1 = after 3 months, T2 = after 6 months, T3 = after 9 months). Note that the time points T0 and T1 include participants from an additional cohort (TC3), explaining the subsequent drop in participant number.

**F - Data of Figure 1 as table**

These tables contain the exact same data as Figure 2. We include them here to facilitate meta-analyses and to alleviate the problem that overlaps make it hard to see the error bars in the Figure.

| **LOCAL POWER AT REST** | | | | | |
| --- | --- | --- | --- | --- | --- |
|  | | **T0** | **T1** | **T2** | **T3** |
| **RCC** | M | 3.934 | 3.896 | 3.816 | 3.852 |
|  | SE | 0.072 | 0.075 | 0.066 | 0.076 |
| **TC1** | M | 3.711 | 3.92 | 3.859 | 3.784 |
|  | SE | 0.072 | 0.067 | 0.068 | 0.078 |
| **TC2** | M | 3.901 | 3.839 | 3.767 | 3.782 |
|  | SE | 0.076 | 0.07 | 0.066 | 0.069 |
| **TC3** | M | 3.863 | 3.907 |  |  |
|  | SE | 0.065 | 0.07 |  |  |

*Table S3a.*

| **LOCAL POWER AT BIOFEEDBACK** | | | | | |
| --- | --- | --- | --- | --- | --- |
|  | | **T0** | **T1** | **T2** | **T3** |
| **RCC** | M | 4.143 | 4.138 | 4.128 | 4.138 |
|  | SE | 0.067 | 0.081 | 0.064 | 0.078 |
| **TC1** | M | 3.916 | 4.151 | 4.139 | 4.008 |
|  | SE | 0.077 | 0.067 | 0.072 | 0.075 |
| **TC2** | M | 4.087 | 4.128 | 4.164 | 4.118 |
|  | SE | 0.073 | 0.07 | 0.069 | 0.068 |
| **TC3** | M | 4.014 | 4.179 |  |  |
|  | SE | 0.067 | 0.069 |  |  |

*Table S3b.*

| **VOLUNTARY UPREGULATION OF LOCAL POWER – CONTROLLED FOR RESPIRATION** | | | | | |
| --- | --- | --- | --- | --- | --- |
|  | | **T0** | **T1** | **T2** | **T3** |
| **RCC** | M | -0.016 | -0.035 | 0.014 | -0.075 |
|  | SE | 0.125 | 0.108 | 0.112 | 0.107 |
| **TC1** | M | -0.089 | -0.007 | 0.111 | -0.068 |
|  | SE | 0.12 | 0.122 | 0.114 | 0.123 |
| **TC2** | M | -0.14 | -0.021 | 0.294 | 0.135 |
|  | SE | 0.123 | 0.11 | 0.13 | 0.117 |
| **TC3** | M | -0.117 | 0.01 |  |  |
|  | SE | 0.089 | 0.114 |  |  |

*Table S3c.*

| **LOCAL POWER AT REST,**  **ONLY TRAINED PARTICIPANTS, SPLIT BY rs53576 POLYMORPHISM** | | | | | |
| --- | --- | --- | --- | --- | --- |
|  | | **T0** | **T1** | **T2** | **T3** |
| **AA** | M | 3.725 | 3.865 | 3.455 | 3.569 |
|  | SE | 0.113 | 0.123 | 0.132 | 0.129 |
| **AG** | M | 3.865 | 3.966 | 3.924 | 3.856 |
|  | SE | 0.071 | 0.06 | 0.072 | 0.081 |
| **GG** | M | 3.81 | 3.821 | 3.788 | 3.75 |
|  | SE | 0.059 | 0.06 | 0.064 | 0.076 |

*Table S3d.*

| **LOCAL POWER AT BIOFEEDBACK,**  **ONLY TRAINED PARTICIPANTS, SPLIT BY rs53576 POLYMORPHISM** | | | | | |
| --- | --- | --- | --- | --- | --- |
|  | | **T0** | **T1** | **T2** | **T3** |
| **AA** | M | 3.77 | 4.089 | 3.989 | 3.91 |
|  | SE | 0.121 | 0.126 | 0.201 | 0.193 |
| **AG** | M | 4.076 | 4.206 | 4.24 | 4.119 |
|  | SE | 0.07 | 0.06 | 0.074 | 0.08 |
| **GG** | M | 4.015 | 4.125 | 4.109 | 4.049 |
|  | SE | 0.059 | 0.059 | 0.065 | 0.068 |

*Table S3e.*

| **VOLUNTARY UPREGULATION OF LOCAL POWER – CONTROLLED FOR RESPIRATION,**  **ONLY TRAINED PARTICIPANTS, SPLIT BY rs53576 POLYMORPHISM** | | | | | |
| --- | --- | --- | --- | --- | --- |
|  | | **T0** | **T1** | **T2** | **T3** |
| **AA** | M | -0.524 | -0.126 | 0.627 | 0.205 |
|  | SE | 0.183 | 0.181 | 0.219 | 0.22 |
| **AG** | M | -0.009 | -0.032 | 0.2 | 0.036 |
|  | SE | 0.102 | 0.104 | 0.122 | 0.137 |
| **GG** | M | -0.085 | 0.053 | 0.093 | -0.006 |
|  | SE | 0.094 | 0.103 | 0.146 | 0.13 |

*Table S3f.*

**References**

1 Singer, T. *et al.* *The ReSource Project: Background, Design, Samples, and Measurements (2nd ed.)*. (Max Planck Institute for Human Cognitive and Brain Sciences, 2016).

2 Kaufmann, T., Sütterlin, S., Schulz, S. & Vögele, C. ARTiiFACT: a tool for heart rate artifact processing and heart rate variability analysis. *Behav Res* **43**, 1161-1170, doi:10.3758/s13428-011-0107-7 (2011).

3 Berntson, G. G. & Stowell, J. R. ECG artifacts and heart period variability: don't miss a beat! *Psychophysiology* **35**, 127-132 (1998).

4 Bornemann, B., Kok, B. E., Böckler, A. & Singer, T. Helping from the heart: Voluntary upregulation of heart rate variability predicts altruistic behavior. *Biological Psychology* **119**, 54-63 (2016).
